# A case of completely isolated advanced enteric duplication cyst cancer performed partial pancreatectomy

**DOI:** 10.1016/j.ijscr.2018.11.060

**Published:** 2018-11-27

**Authors:** Shinsuke Nakashima, Terumasa Yamada, Go Sato, Takaaki Sakai, Yoshinao Chinen, Hiroaki Itakura, Ryo Kato, Masami Ueda, Yujiro Tsuda, Katsuya Ohta, Jin Matsuyama, Masakazu Ikenaga

**Affiliations:** Department of Gastroenterological Surgery, Higashiosaka City Medical Center, Nishiiwata 3-4-5, Higashiosaka, Osaka, 567-8588, Japan

**Keywords:** CT, computed tomography, CE-CT, contrast enhanced computed tomography, DWI, diffusion weighted image, MRI, magnetic resonance imaging, MCN, mucinous cystic neoplasm, IPMN, intraductal papillary mucinous neoplasm, T1WI, T1 weighted image, T2WI, T2 weighted image, Enteric duplication cyst, Duplication cyst cancer, Pancreatic cyst

## Abstract

**Introduction:**

Enteric duplication cysts are rare and, in addition, isolated enteric duplication cysts are lower morbidity prevalence rate. These cysts lack a connection to the gastrointestinal tract or the adjacent mesenteric vasculature and have only been reported in 10 case reports. In these reports, only two reports were cases with malignant transformation. Our case was a report for the advanced cancer of the isolated enteric duplication cyst.

**Case presentation:**

The patient was a 43 year-old woman with slightly abdominal pain and mass formation. The abdominal contrast-enhanced computed tomography showed 130 × 100 × 90 mm huge cystic mass existed in right upper peritoneal cavity. The cystic mass had thickened wall and many enhanced nodules. As these imaging findings suggested a tumor originated from pancreas and the preoperative diagnose was suspect of mucinous cystic neoplasm. In operative findings, the tumor originated from pancreatic head and did not attach to gastrointestinal tract. Final pathology indicated the cyst was an isolated advanced enteric duplication cyst cancer and not originated from pancreas.

**Conclusion:**

We experienced an extremely rare case of completely isolated advanced enteric duplication cyst cancer. Unique to this case, the preoperative diagnosis was suspect of mucinous cystic neoplasm arising from pancreas head and partial pancreatectomy was performed. However, in the pathological findings, this cyst diagnosed advanced enteric duplication cyst cancer.

## Highlights

• Duplication cysts commonly have connection to the gastrointestinal tract but the cysts are rarely isolated from gastrointestinal tract.
• Malignant transformation in isolated duplication cysts is extremely rare.
• This case of advanced cancer with the isolated intestinal duplication cyst was second reported worldwide.
• The preoperative diagnosis was suspect of mucinous cystic neoplasm arising from pancreas head and partial pancreatectomy was performed. However, in the pathological findings, this cyst diagnosed advanced enteric duplication cyst cancer and not originated from pancreas.

## 1 Introduction

Enteric duplication cysts are rare congenital malformation that originate anywhere along the alimentary tract. The more than half of the cases are diagnosed early childhood due to symptoms, abdominal pain, gastrointestinal bleeding, intestinal obstruction, abdominal mass, and so on [[1], [2], [3]]. Duplication cysts commonly have connection to the gastrointestinal tract but the cysts are rarely isolated from gastrointestinal tract [4].

Malignant transformation in isolated duplication cysts is extremely rare. Here we reported a resected case of advanced cancer arising from an isolated enteric duplication cyst surrounding the pancreas that initially diagnosed a mucinous cystic neoplasm (MCN).

This report is a work based on Consensus-based surgical case report guidelines, SCARE criteria [5].

## 2 Case presentation

A 43 year-old female was admitted to our hospital with a chief complaint of right upper pain and palpable mass. On physical examination, there was slight tenderness and semimobile mass in the right upper abdomen. Clinical examination did not demonstrate any persistent weak abdominal pain and mass. Her past medical and surgical history was unremarkable. Moreover, Family history and psychosocial history were also not particular.

**Fig. 1:**
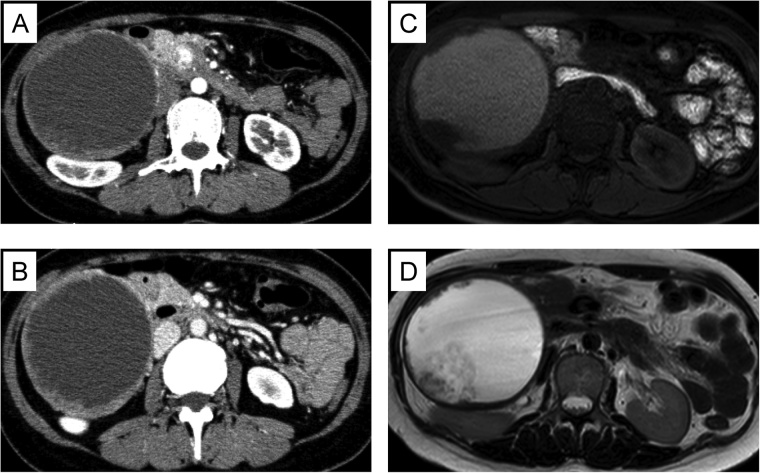
Imaging findings in CE-CT and MRI. CT showed that the huge cyst had thickened wall and many enhanced nodules. (A) Early phase. Many nodules and the thickened wall were slightly enhanced. (B) Delay phase. They were gradually enhanced. MRI showed that the intensity of intra-cystic fluid was slightly high in T1WI (C) and high in T2WI (D).

In abdominal contrast-enhanced computed tomography (CT), 130 × 100 × 90 mm huge cystic mass was demonstrated in right upper peritoneal cavity. The cyst had thickened wall and many enhanced nodules. Many nodules and the thickened wall were slightly enhanced in early phase (Fig. 1A) and gradually enhanced in delayed phase (Fig. 1B). Gadolinium-enhanced magnetic resonance imaging (MRI) showed that intra-cystic fluid was bleeding or mucinous fluid as the intensity was slightly high in T1WI (Fig. 1C), high in T2WI (Fig. 1D), and slightly high in fat suppression T1WI. The thickened wall of the cyst and mural nodules were high intensity in Diffusion-weighted MRI. The findings of connection with pancreatic head suggested a tumor originated from pancreas. The differential diagnoses were MCN, mucinous cystic adenocarcinoma and hemorrhagic cyst. After an extensive discussion in our institute, we planned pancreaticoduodenectomy for this abdominal tumor.

**Fig. 2:**
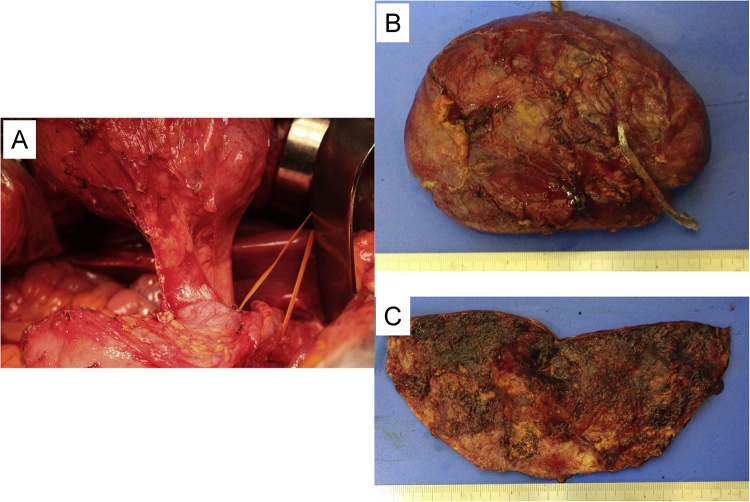
Surgical findings. (A) The cyst did not attach to gastrointestinal tract and originated from pancreatic head. (B) Resected specimen: The cyst was resected completely by partial pancreatectomy. (C) The cystic wall was thickened and elastic soft.

She was taken to the operating room by a surgical oncologist who primarily specialized in pancreatic resections and had been in practice for over 10 years. In surgical findings, this tumor did not attach to gastrointestinal tract and originated from pancreatic head (Fig. 2A). The aspirated fluid was bloody and the cyst had re-increased for short time in operation. In these reasons, we diagnosed the tumor as hemorrhagic cyst and selected partial pancreatectomy because this tumor was resected completely (Fig. 2B). The cystic wall was thickened and elastic soft. No tumoral change found in the surface of cystic wall (Fig. 2C). Post-operative course was good and she was discharged on post-operative day 9. CEA and CA19-9 in intra-cystic fluid were 115,060 ng/ml and 113,373 U/ml.

**Fig. 3:**
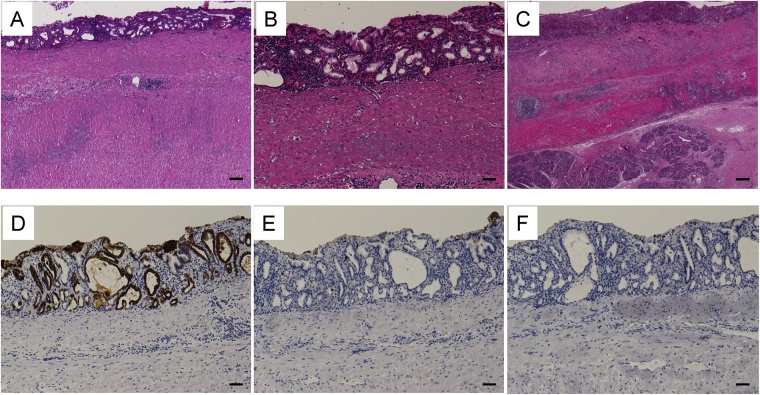
The pathological findings of non-tumoral tissue (A–C). Non-tumoral epithelium was similar to crypt epithelium of the stomach. The immunohistochemistry was positive for CK7 (D), negative for CK20 (E), and negative for CDX-2 (F). Bar = 100 μm.

**Fig. 4:**
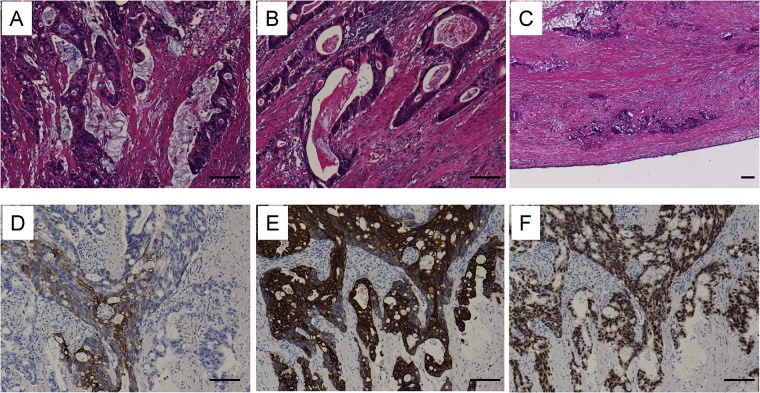
The pathological findings of tumoral tissue (A–C). Adenocarcinoma with moderate to well differentiation invaded to subserosal layer in broad area of the cystic wall. The immunohistochemistry of the tumoral mucosa was 50% positive for CK7 (D), positive for CK20 (E), and positive for CDX-2 (F). Bar = 100 μm.

Final pathology demonstrated that the cystic mass had well-formed cyst wall with an inner mucosal lining, submucosal layer, and muscularis propria as gastrointestinal tract (Fig. 3A–C). As the non-tumoral epithelium in small area was similar to crypt epithelium of the stomach and immunohistochemistry of the non-tumoral mucosa was positive for CK7 (Fig. 3D), negative for CK20 (Fig. 3E), and negative for CDX-2 (Fig. 3F), the pathological finding of non-tumoral tissue was the enteric duplication cyst of gastric type. Adenocarcinoma with moderate to well differentiation invaded to subserosal layer over smooth muscle layer in broad area of the cystic wall (Fig. 4A–C). The invasive cancer cells invaded lymphatic system, venous system, and nervous system. Several lymph nodes resected together besides tumor had no metastasis. The immunohistochemistry of the tumoral mucosa was 50% positive for CK7 (Fig. 4D), positive for CK20 (Fig. 4E), and positive for CDX-2 (Fig. 4F). The final diagnosis was completely isolated advanced enteric duplication cyst cancer because this cyst was not attached to a wall of gastrointestinal tract and adjacent to pancreatic head. Therefore, this cyst was not originated from pancreas head.

During hospital stay in post-operative period, she was counseled that we identified an isolated enteric duplication cyst with advanced cancer. We recommend treatment by S-1 as adjuvant chemotherapy according to the adaptation for criteria of advanced gastric cancer. She had taken S-1 for 1 year without rest of medication and lived for 1.5 year after surgery without any evidence of malignancy.

## 3 Discussion

Intestinal duplication cyst are rare, seen in approximately 1/100,000 births [6,7]. These cysts can arise from the gastrointestinal tract and have a predilection for the jejunum and ileum [1,8,9]. In rare cases, the duplication cyst can be completely isolated from gastrointestinal tract. These cases were characterized in 10 reports and one case was an isolated intestinal duplication cyst surrounding the pancreatic body [9,10] (Table 1). Malignant formation was reported in only two cases. One was retroperitoneal duplication cyst seen as carcinoma in situ and the other was poorly differentiated tubular adenocarcinoma invading muscularis propria in mid abdominal cavity [11,12]. Our case of advanced cancer with the isolated intestinal duplication cyst was second reported.

**Table 1:** Total reported cases of isolated enteric duplication cyst.

References	year	Age	Gender	Clinical feature	Size (cm)	Site	Mucosal type	Malignancy
Kim et al. [14]	2003	28	M	Incidental	Not mentioned	Mesentery of ligament of Treitz	Gastric	No
Lee et al. [15]	2010	21	F	Palpable mass	3.5 × 2.5	Mesentery of jejunum	No epithelial lining	No
Nichols et al. [16]	2011	27	F	Abdominal fullness	9 × 4 × 1	Mesentery of descending colon	Simple columnar epithelium	No
Metehan et al. [17]	2011	28	M	Abdominal pain and palpable mass	25 × 6	Mesentery of ileum	Not mentioned	No
Blank et al. [12]	2012	51	M	Incidental	10 × 4	Mesentery of ileum	Vilii, crypts, numerous mucous cells	Por
Pant et al. [18]	2012	1	M	Abdominal pain and distension	8	Mesentery of ileum	Gastric	No
Kyriakos et al. [19]	2013	20	M	Abdominal pain and fever	7 × 4	Lateral region of Ascending colon	Not mentioned	No
Park et al. [13]	2014	36	F	Abdominal pain	12 × 8.5 × 6	Mesentery of ileum	Mixed	No
Weitman et al. [10]	2017	48	F	Abdominal pain	6.5 × 4.5 × 2.5	Peripancreas	Jejunal	No
Faraji et al. [11]	2017	64	F	Abdominal pain and fatigue	6.9 × 6.6 × 6.1	Retroperitoneum	Columnar epithelium with high grade dysplasia	CIS
Our case	2018	43	F	Abdominal pain and palpable mass	13 × 10 × 9	Peripancreas	Gastric	Tub2 > pap

The differential diagnoses for intra-abdominal cystic tumor around pancreas are MCN, pancreatic pseudocyst, mesenteric and omental cysts, and so on. In imaging modality, the cystic wall indicated thickened and enhanced partially. As the intra-cystic fluid had seen serous or bloody, the density and intensity were various. Therefore, the definitive diagnosis was difficult and this case was diagnosed with MCN. CEA and CA19-9 level in intra-cystic fluid was often high in MCN and IPMN and it was unuseful to distinguish malignancy [13].

Histologically, in stricture and histomorphology, enteric duplication cysts are similar to normal bowel wall and consist of mucosa, submucosa, muscularis propria and serosa. The mucosal type is reported as gastric and jejunal type by thickness of mucosa and submucosa and structure formation of villi and crypt [8].

As it was not reported treatment for an advanced isolated duplication cyst cancer, there is no standard treatment and surgical resection have been only managed. This case was advanced cancer and we have treated her by adjuvant chemotherapy, S^-1^ (100 mg/day, 6 week/cycle, 1 year), as advanced gastric cancer. No standard treatment has been established for advanced isolated duplication cyst cancer because of its rarity and variable extent of this disease, and the management would be performed based on one of gastrointestinal tract cancer for the time being.

This case was second report of the isolated intestinal duplication cyst with advanced cancer and performed R0 resection. The work indicated the findings of clinical examinations, pathology, and course of treatment.

## 4 Conclusion

We experienced an extremely rare case of completely isolated advanced enteric duplication cyst cancer. Unique to this case, the preoperative diagnosis was suspect of mucinous cystic neoplasm arising from pancreas head and partial pancreatectomy was performed. However, in the pathological findings, this cyst diagnosed advanced enteric duplication cyst cancer.

## Conflicts of interest

All authors have no conflicts of interest.

## Sources of funding

All authors declare no sources of funding. I state in the text (page 7).

## Ethical approval

This study of case report is exempt from ethnical approval in ethics committee of our institution.

## Consent

This manuscript includes a statement to this effect in a consent section at the end of the manuscript.

Patient’s names, initials, or hospital numbers is not used. Identified information in the images of the patient is not also used and she has a right to privacy.

## Author contribution

Clinical treatment: Shinsuke Nakashima, Terumasa Yamada, Go Sato, Takaaki Sakai, Yoshinao Chinen, Hiroaki Itakura, Ryo Kato, Masami Ueda, Yujiro Tsuda, Katsuya Ohta, Jin Matsuyama, and Masakazu Ikenaga.

Collected data: Shinsuke Nakashima, Yujiro Tsuda and Terumasa Yamada.

Assessment and discussion: Shinsuke Nakashima and Terumasa Yamada.

Wrote the paper: Shinsuke Nakashima and Terumasa Yamada.

## Registration of research studies

We confirm that the work is not necessary to registry UIN. This work was not included its first in man ie the first time a new device or surgical technique. The report of one case is not necessary to ethnical approval in our institution.

## Guarantor

The guarantor of this study is Terumasa Yamada, corresponding author.

## Provenance and peer review

Not commissioned, externally peer reviewed.
